# Autosomal Recessive Becker’s Form of Myotonia Congenita in Indian Families

**DOI:** 10.7759/cureus.84373

**Published:** 2025-05-18

**Authors:** Sahithi Krovvidi, Srilaxmi Nelakurthi, Mounika Gedela, Srinithya Singamsetty, Vijayalakshmi Bhimireddy

**Affiliations:** 1 Internal Medicine, NRI Medical College and General Hospital, Guntur, IND; 2 Pediatrics, NRI Medical College and General Hospital, Guntur, IND

**Keywords:** becker’s disease, clcn1 gene, mutation, myotonia congenita, non-dystrophic myotonia

## Abstract

Myotonia congenita (MC), a rare inherited disease, is caused by variations in the skeletal muscle chloride voltage-gated channel one gene (*CLCN1*) and is characterized by symptoms of myotonia and muscle hypertrophy. We present a case report of two female patients aged nine and 10, from Andhra Pradesh, India, with a history of parental consanguinity, hypertrophy of arm and calf muscles, permanent weakness, and proximal muscle weakness. Patients were diagnosed with Becker’s form of MC after genetic testing that reported the mutation c.1667T >A (p.lle556Asn) in exon 15 of the *CLCN1*, which is a pathogenic variant. Treatment with mexiletine showed improvement in the condition of patients. Because of its inherent nature, parents were given genetic counseling and the choice of antenatal diagnosis for upcoming pregnancies.

## Introduction

Myotonia congenita (MC) is an inherited disease that develops gradually between 4 and 14 years of age and is characterized by the symptoms of painless myotonia beginning from lower limbs and spreading to the muscles of arms, neck, and face, and generalized muscle hypertrophy, which gives rise to the bodybuilder like shape, generally referred as Herculean appearance [1]. Disease is rare, and the reported incidence is 0.3 to 0.6 per one lakh people globally [2]. Disease is due to a mutation of the skeletal muscle chloride voltage-gated channel one (*CLCN1*) gene located at chromosome 7q35 [3] and can be either dominant (Thomsen disease) or recessive (Becker disease). Mutations in the *CLCN1* gene cause impaired muscle relaxation after a contraction leading to stiffness, cramping, and muscle rigidity. Affected individuals have abnormal enlargement of muscles, a bodybuilder appearance [2]. Consanguinity increases the probability of MC in children because of the recessive state of the gene. The clinical severity is linked to the genotype, the recessive form being the severe and widespread. In general, the disease is diagnosed through clinical signs and validated by genetic testing. The majority of cases of Becker’s form of MC have been reported in European people [4,5], with a high percentage in northern Europe [6]. Only a few cases were reported in other populations including India [7-11]. This case report presents Becker’s form of MC in two Indian families, featuring unique genetic and clinical findings. ​ Understanding the pathophysiology, diagnosis, and treatment of MC is crucial for improving patient outcomes and raising awareness of this rare condition.​

## Case presentation

Case 1

**Figure 1:**
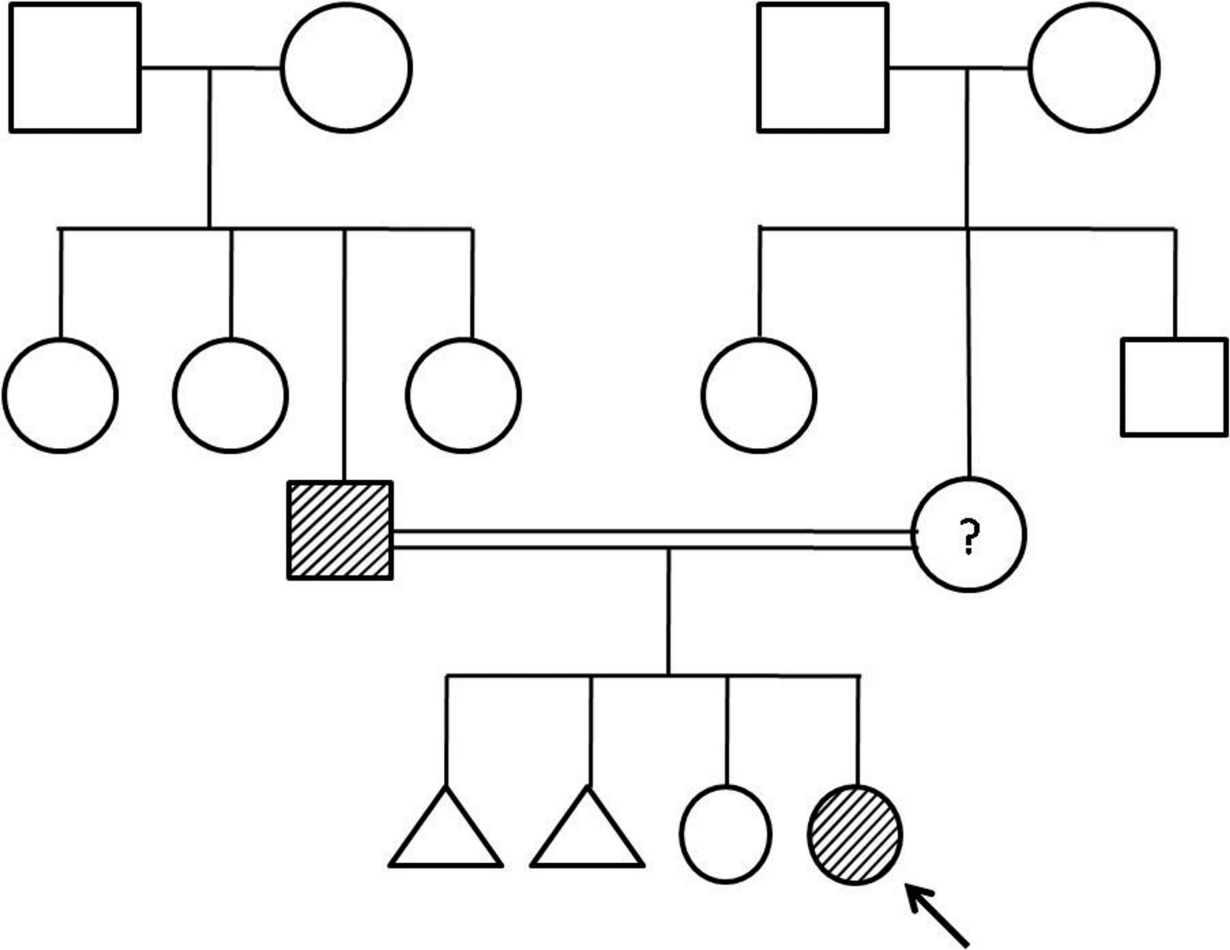
Pedigree of the proband The arrow indicates the female proband

A nine-year-old girl born to third-degree consanguinity parents, weighing 30.0 kg, presented with difficulty getting up from sitting posture, leg cramps after prolonged walking, and frequent falls, suggestive of proximal muscle weakness. Symptoms began at age four. Thereafter, she experienced difficulty getting up from a sitting position and needed hand support. Her father, a laborer, had similar complaints but to a lesser degree, with no restriction in daily activities (Figure 1). The affected child's (indicated by the arrow) father is assumed to be affected by history and the mother is a suspected carrier.

Case 2

A 10-year-old girl born to second-degree consanguinity parents (maternal uncle-niece) and second in the birth order was presented with similar symptoms like difficulty getting up from a sitting posture and frequent falls. Dysmorphic facies with hypertelorism was noted. There is no report of a similar complaint among other family members.

**Figure 2:**
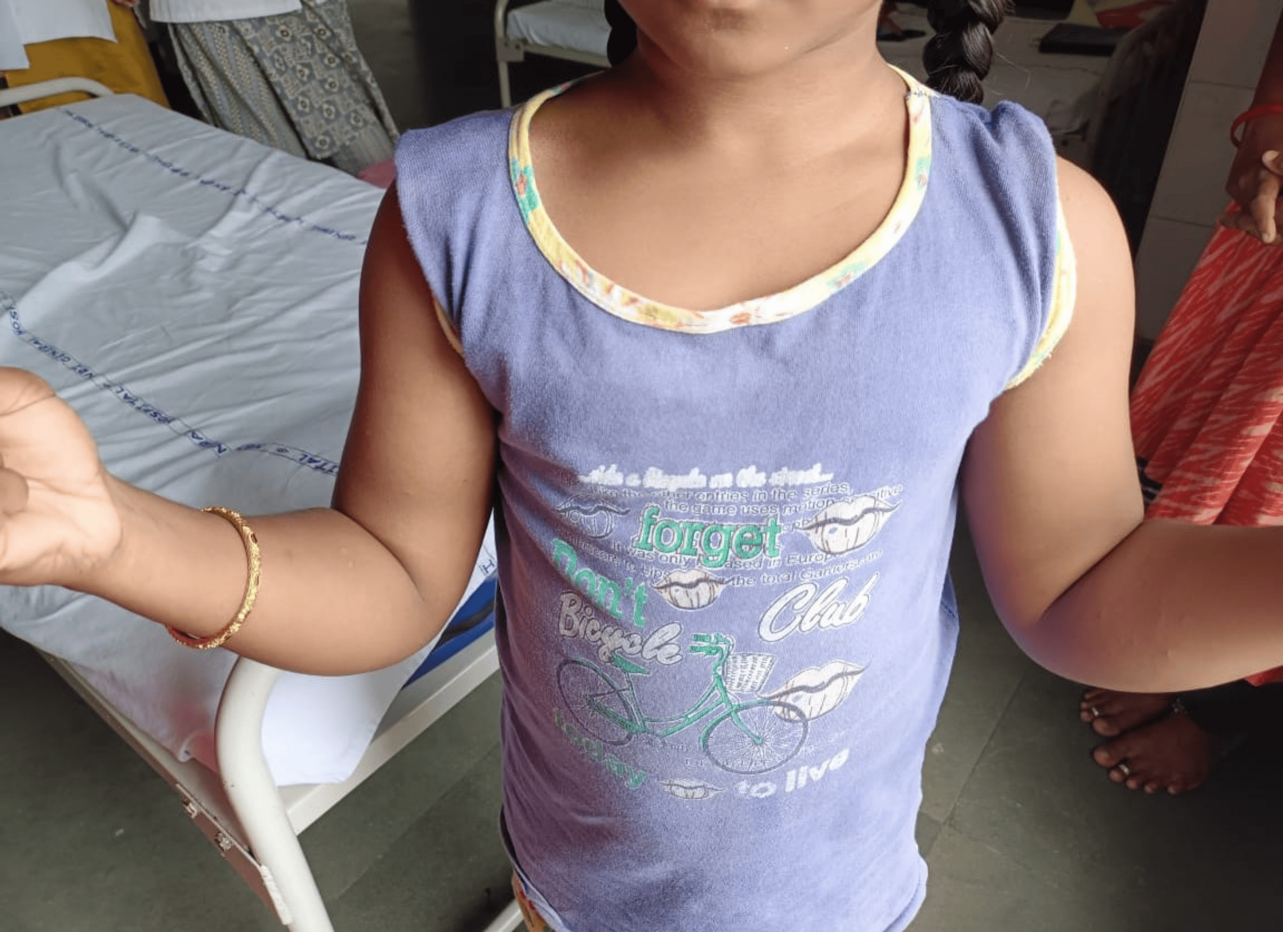
Hypertrophy of shoulder muscles (case 1) Bodybuilder appearance due to prominent shoulder girdle muscles indicative of Becker’s form of MC MC: Myotonia congenita

**Figure 3:**
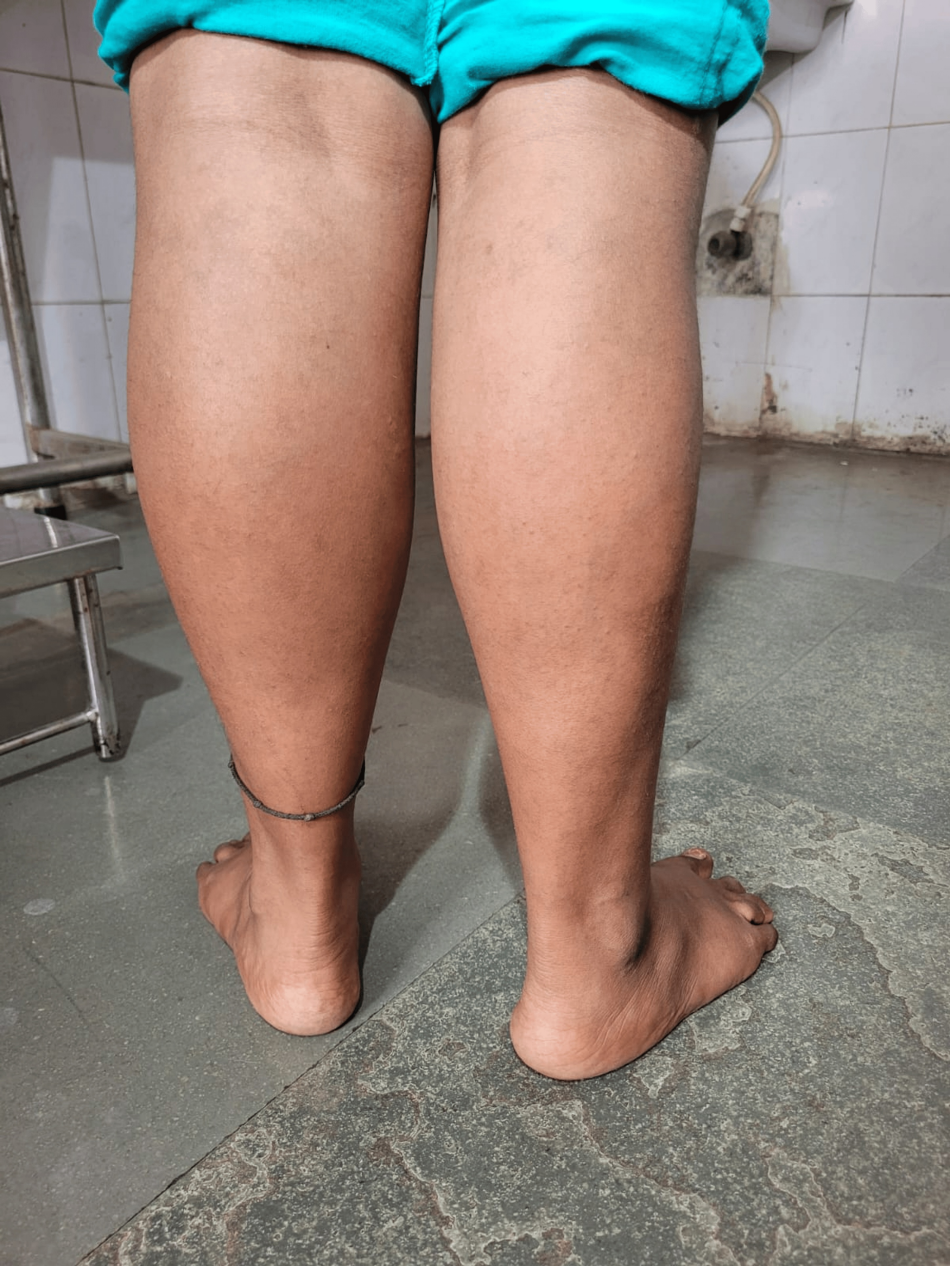
Hypertrophy of calf muscles (case 2) Figure showing the prominent calf muscle hypertrophy

Both children had easy fatigability for outdoor games and initially approached several hospitals. Vitamin D3 and calcium supplementation was evident as per history, which did not alleviate the symptoms over the period. However, in both cases, the symptoms did not coincide with loss of sensation. In addition, there are no neurocutaneous markers. Following examination, the girl's vitals were found to be stable. Hypertrophy was noticed in their upper and lower limbs (shoulder and calf muscles) (Figures 2, 3), with Herculean appearance and negative Grower's sign.

There was decreased strength in extremities, measuring 4 out of 5. The absence of Grower's sign indicates that the patient does not require hand support to rise from a sitting position, differentiating this condition from other neuromuscular disorders.

Musculoskeletal system examination of girls indicated that muscle relaxation was improved on stimming. Percussion myotonia was elicited in the tongue (Video 1), calf muscles, and thenar muscles.

**Video 1:** Percussion myotonia - tongue (case 2)

Percussion myotonia with diminished deep tendon reflexes (DTRs) without cortical, sensory and cerebellar involvement was noticed in the eight-year-old girl and normal DTRs in the 10-year-old girl. Hand grip myotonia was observed (Video 2).

**Video 2:** Hand grip myotonia (case 2) Handgrip myotonia exhibiting delay in relaxing of the palm after a sustained grip.

Other systemic examinations were not remarkable (Table 1).

**Table 1:** Clinical features of patients with MC MC: Myotonia congenita; CLCN1: chloride voltage-gated channel 1 gene

Characteristics	Case 1	Case 2
Sex	Female	Female
Age onset (yr)	4	9
Family history	Yes	No
Consanguinity	3	2
Inheritance	Autosomal recessive	Autosomal recessive
Clinical/Percussion myotonia		
Tongue	Present	Present
Hands	Present	Present
Legs	Present	Present
Grip Myotonia	Present	Absent
Triggers		
Exercise	Present	Absent
Cold	Present	Present
Fatigue	Present	Present
Other observations		
Dysmorphic facies	Absent	Present
Muscle hypertrophy	Present	Present
Muscle pain	Present	Absent
Permanent weakness	Present	Present
CLCN1 mutation	p.lle556Asn	p.lle556Asn
Diagnosis	Becker's form of MC	Becker's form of MC

Gait was normal. Blood parameters were normal except for serum creatine kinase, which is elevated (Table 2).

**Table 2:** Laboratory investigations TSH: Thyroid-stimulating hormone, Ft4: free thyroxine; ALP: alkaline phosphatase; FBS: fasting blood sugar; CPK: creatine phosphokinase

	Case 1	Case 2	Reference values
Complete blood profile			
Hb (gm/dl)	12.7	13.3	11.5-15.5
WBC (cells/cumm)	8250	6600	4500-11000
Platelets (millions/mcL)	0.278	0.28	0.15-0.40
Thyroid profile			
Ft4 (ng/dL)	1.2	1.5	0.8-1.9
TSH (mIU/L)	3.61	2.43	0.4-4.0
Bone profile:			
Serum phosphorus (mg/dL)	3	3.9	2.5-4.5
Serum calcium (mg/dL)	9	10	8.5-10.2
ALP (IU/L)	114	131	30-130
Vit-D (ng/mL)	50	31	30-100
FBS (mg/dL)	84	91	<100
Liver function tests	Normal	Normal	-
Renal function tests	Normal	Normal	-
CPK (U/L)	154	184	30-135
2D-Echo	Normal	Normal	-
ECG	Normal	Normal	-

For both cases, the EMG was not done as it is invasive and involves voluntary contraction by the child; moreover, it is not confirmatory. Hence, diagnosis was established by subjecting the blood sample of patients to DNA isolation and gene sequencing (MedGenome Labs Ltd, Bengaluru). Genetic testing is important to confirm the diagnosis and to establish the pattern of inheritance. Genetic testing reported a homozygous state due to c.1667T>A in exon 15 of the *CLCN1* that encodes the ClC-1 protein, the skeletal muscle chloride channel protein responsible for the myotonia congenita. Being a genetic disorder, it does not have a complete cure. Though non-pharmacological interventions are advantageous for therapy, they demand pharmacological interventions in severe cases. The girls were treated with mexiletine at 200 mg three times daily for three weeks, which improved stiffness and quality of life in patients and was well tolerated. Patients were also advised to undergo physiotherapy. Because of its inherent nature, parents were given genetic counseling and the choice of antenatal diagnosis for upcoming pregnancies.

## Discussion

MC, a type of non-dystrophic myotonia, is a rare genetic muscle disorder defined by hypertonia and myotonia and manifests during the later stages of childhood, typically between 6 and 12 years of age. It is specified by episodes of myotonic stiffness and alleviated by exercise (warmup effect) and percussion myotonia. In general, the disease is diagnosed through clinical signs, electromyography, muscle biopsy, and genetic analysis. Earlier, electromyography was fundamental in the diagnosis of this disease, but it has now been replaced by genetic analysis. Limited availability or absence of medical geneticists and genetic testing could be the reason for the lower incidence of this disorder. In the current study, the two girls presented a slight clinical variability. Both patients presented with proximal muscle weakness, frequent falls, and difficulty getting up from a sitting posture. ​ Muscle hypertrophy was observed in the shoulder and calf muscles, contributing to the Herculean appearance characteristic of Becker’s form of MC. The symptoms were aggravated by cold weather and exercise. Elevated serum creatine phosphokinase levels (154 U/L in case 1 and 184 U/L in case 2) suggest muscle damage, consistent with the clinical presentation. Normal thyroid, liver, and renal function tests ruled out other systemic causes of muscle weakness. Genetic testing identified a homozygous mutation c.1667T>A (p.lle556Asn) in the *CLCN1* gene, establishing MC. The genotype affects phenotype, and the mode of transmission of this gene variant observed through the pedigree chart will comprehend the risk of disease transmission and development of the disease in the families.

This transversion mutation was classified as pathogenic [12] and linked to severe clinical manifestations. It has also been reported in a 50-year-old Malaysian woman with muscle stiffness [13]. The resultant p.I556N due to T→A leads to serious voltage dependence. However, the patients with a heterozygous state at the locus exhibit milder symptoms due to a trivial shift [14]. It is hypothesized that the father of case 1, who had similar symptoms with a lesser degree, could be heterozygous. Similar observations were also reported by Plassart-Schiess et al. and Arzel-Hézode et al. [15,16]. The mutations of the *CLCN1* gene result in a decrease in channel opening probability and make the membrane excitable, manifesting myotonia. So far, a number of (>318) pathogenic variants in the *CLCN1* gene have been identified in human populations [13], the majority of which are linked to the Becker form, and about 27 mutations with the Thomsen form [17]. This condition is differentiated from paramyotonia congenita by the lack of hyperkalemic periodic paralysis and sodium channel myotonia, with the absence of eyelid myotonia.

Mexiletine at 200 mg three times daily improved stiffness and quality of life in both patients. The medication is continued with periodical evaluation as an outpatient. Though several pharmacological interventions, namely carbamazepine, mexiletine, and phenytoin, have been used for MC, currently, mexiletine is the only FDA-approved drug to address the MC [18]. Certain medications, such as depolarizing muscle relaxants (e.g., suxamethonium), beta-adrenergic agonists, and adrenaline, may exacerbate myotonia and, thus, are contraindicated in children. Patients were also suggested lifestyle changes such as small, frequent meals, avoiding fatty and spicy foods, and ensuring upright posture after eating to avoid indigestion-reflux, which is a common complication in mexiletine use as per the recent report [19]. In addition to treatment, physiotherapy and lifestyle changes were recommended to enhance long-term outcomes. ​ Parents were counseled on genetic risks and antenatal diagnosis for future pregnancies.

## Conclusions

In these two cases, a typical Herculean appearance with myotonia and warm-up phenomenon clearly indicates MC. Genetic analysis identified a rare homozygous c.1667T>A (p.lle556Asn) pathogenic variant in the *CLCN1* gene, establishing Becker’s type of MC. This variant manifested autosomal recessive inheritance. Mexiletine had a promising role in disease control. Understanding the pathomechanism is necessary for appropriate diagnosis, genetic counseling of patients, and better medical interventions. Sensitizing healthcare professionals on MC and further epidemiological studies can aid in recognizing undiagnosed myotonia cases in the country.
